# Mechanistic Insights into Chemoresistance Mediated by Oncogenic Viruses in Lymphomas

**DOI:** 10.3390/v11121161

**Published:** 2019-12-16

**Authors:** Jungang Chen, Samantha Kendrick, Zhiqiang Qin

**Affiliations:** 1 Department of Pathology, Winthrop P. Rockefeller Cancer Institute, University of Arkansas for Medical Sciences, 4301 W Markham St, Little Rock, AR 72205, USA; jchen2@uams.edu (J.C.); skendrick@uams.edu (S.K.); 2 Department of Biochemistry and Molecular Biology, Winthrop P. Rockefeller Cancer Institute, University of Arkansas for Medical Sciences, 4301 W Markham St, Little Rock, AR 72205, USA

**Keywords:** chemoresistance, lymphoma, oncogenic virus, EBV, KSHV

## Abstract

Viral lymphomagenesis induced by infection with oncogenic viruses, such as Kaposi’s sarcoma associated herpesvirus (KSHV), Epstein–Barr virus (EBV) and human T-cell leukemia virus (HTLV-1), represents a group of aggressive malignancies with a diverse range of pathological features. Combined chemotherapy remains the standard of care for these virus-associated lymphomas; however, frequent chemoresistance is a barrier to achieving successful long-term disease-free survival. There is increasing evidence that indicates virus-associated lymphomas display more resistance to cytotoxic chemotherapeutic agents than that observed in solid tumors. Although the tumor microenvironment and genetic changes, such as key oncogene mutations, are closely related to chemoresistance, some studies demonstrate that the components of oncogenic viruses themselves play pivotal roles in the multidrug chemoresistance of lymphoma cells. In this review, we summarize recent advances in the understanding of the mechanisms through which oncogenic viruses mediate lymphoma cell chemoresistance, with a particular focus on KSHV and EBV, two major oncogenic viruses. We also discuss the current challenges to overcome these obstacles in the treatment of virus-associated lymphomas.

## 1. Introduction

Virus-associated lymphomas represent a largely heterogeneous group of hematologic malignancies that are characterized by the uncontrolled growth of clonal lymphocytes [1]. The pathogenesis of virus-associated lymphomas is a complex process involving viral infections, autoimmune diseases, environmental and genetic factors, and exposure to chemical or other toxins [2,3]. Extensive published literature show a close interrelation between lymphomagenesis and infection by viruses, including Kaposi’s sarcoma associated herpesvirus (KSHV) [4], Epstein–Barr virus (EBV) [5], hepatitis C virus (HCV) [6], human T-cell leukemia virus (HTLV) [7], and human immunodeficiency virus (HIV) [8,9]. Interestingly, Dr. Robert Gallo’s group provided new evidence that some HIV-1 matrix protein variants (vp17s) can activate AKT signaling and promote growth of transformed B cells [10]. Moreover, vp17s are more frequently detected in plasma of HIV+ patients with non-Hodgkin’s lymphoma (NHL) compared to those patients without NHL [10]. These data taken together with other recent findings, including the persistence of HIV-associated proteins within lymphoid follicles, indicates a direct role of HIV-1 in promoting lymphomagenesis beyond immune suppression functions [11]. However, the HIV-1 genome is not detectable in these malignant B cells and the transforming ability of HIV-1 in B cells still needs further investigation. Thus, this review will focus on the two major oncogenic viruses that cause B-cell, T-cell, and NK-cell lymphomas: KSHV and EBV.

KSHV and EBV both belong to the γ-herpesviridae subfamily and exhibit two alternative life cycle programs during infection of host cells: The latent and lytic phases. Although there is accumulating data that collectively support the involvement of both latent and lytic programs in the development and maintenance of viral diseases [12,13], the latent transcripts may play a more crucial and prominent role in tumorigenesis [14]. The ability for prolonged latent infection increases the risk for cancer development, and until last year, there was little known about the mechanism behind the maintenance of persistent latency and prevention of the lytic phases. Suppression of genes that induce the lytic phase in both KSHV- and EBV-infected cells in culture and from infected patients can occur through Krüppel-associated box (KRAB) domain-zinc finger protein transcriptional repressors, stem cell zinc finger protein 1 (SZF1) and zinc finger protein 557 (ZNF557), which are both regulated by STAT3, revealing a new epigenome regulatory activity of STAT3 [15]. The less than ideal effectiveness of antiviral agents against lytic phases achieved in the clinic suggests there are additional, intractable functions of herpesvirus latent infection during treatment [16,17,18]. During the latency of KSHV, only a limited number of viral proteins are expressed, including Latency associated nuclear antigen (LANA), LANA-2, viral Cyclin (vCyclin), viral Fas-associated death domain-like interleukin-1β-converting enzyme-inhibitory protein (vFLIP), Kaposins, and at least 18 mature viral microRNAs [19]. Of note, some of these latent components are oncogenic and involved in immune escape and lymphomagenesis [20]. More recently, KSHV-encoded IL-6 was added to this list as B-cells in mice infected with KSHV were shown to upregulate activation-induced cytidine deaminase (AID) expression, an enzyme responsible for class switch recombination for antibody generation, that correlated with vIL-6 and an increase in class switching [21]. With AID implicated in misappropriated translocations and mutations in lymphoma, vIL-6 may also play a role in KSHV pathogenesis and the development of KSHV-lymphoproliferative disorders and primary effusion lymphoma (PEL), the main type of KSHV-associated lymphoma. PEL is a rare but rapidly progressive large B cell lymphoma clinically characterized by malignant effusion in body cavities usually without extracavitary tumor masses [22]. Approximately half of PEL patients have pre-existing or develop Kaposi’s sarcoma (KS, another cancer caused by KSHV) [23]. PEL is most commonly found in persons living with HIV and other immunocompromised individuals [24]. Interestingly, KHSV–EBV co-infection is also commonly found (60%–90% of cases) in PEL [25], although the role of this simultaneous infection in the pathogenesis of PEL remains unclear.

In contrast to the persistent latency of KHSV, EBV infection has three distinct latency patterns: Latency I, II, and III due to the expression of specific latent proteins seen in related diseases and/or cell lines, including EBNA1 (Epstein–Barr nuclear antigen 1), EBNA2, EBNA3A/3B/3C, LMP1/2, and some EBV-encoded small RNAs (EBERs) [26,27,28]. Latency states of EBV are identified according to the development of EBV-related tumors. EBV is considered as the epitome of a B lymphotropic virus, and as such, its infection is closely associated with various types of lymphomas: Classical Hodgkin lymphoma (cHL), Burkitt’s lymphoma (BL), T-cell/NK-cell lymphoma, diffuse large B-cell lymphoma (DLBCL), AIDS-associated lymphoma, post-transplant lymphoma (PTLD), and other rare B-cell and T-cell lymphoproliferative disorders [5,29,30]. There are different patterns of viral gene expression within these lymphomas. For instance, BL is always associated with latency I infection characterized by EBNA1 expression [31]. Latency II is associated with the expression of EBNA1, LMP1, and LMP2, usually seen in HL and T-cell NHL [32,33,34]. In contrast, the expression of all these latent components is present in latency III, occurring mainly in immunocompromised patients suffering from PTLD, AIDS-associated lymphoma, or in some lymphoblastoid cell lines [35].

Therapeutic regimens for virus-associated lymphomas include radiotherapy, chemotherapy, highly active antiretroviral therapy, antiviral agents, targeted therapy, specific cytotoxic T-cells, and immunotherapy [17,18,22,25,36,37]. Among these, conventional and cytotoxic chemotherapy is still the standard of care for most virus-associated lymphomas. However, most virus-associated lymphomas usually display higher rates of chemoresistance compared to solid tumors. Although there are complex factors that contribute to the chemoresistance of virus-associated lymphomas, the intrinsic viral proteins or components play a pivotal role in the chemoresistant phenotype of tumor cells. For instance, recent efforts to target EBNA1 with small molecules and inhibit its DNA binding activity proved useful in suppressing tumor growth in lymphoblastoid and patient-derived nasopharyngeal carcinoma models [38]. Resolving the mechanisms through which oncogenic viruses mediate chemoresistance may bring new insights and promising strategies to improve chemotherapy efficacy and allow for dose reduction to minimize the toxicity of chemotherapeutic agents in cancer patients. In this article, we will review the current advances in chemotherapeutic treatment for virus-associated lymphomas and the findings of oncogenic viral protein-mediated chemoresistance.

## 2. Current Status of Chemotherapy in Virus-Associated Lymphomas

### 2.1. KSHV-Associated Lymphoma

As mentioned above, PEL represents the major lymphoma etiologically related to KSHV. The prognosis for PEL remains poor, with a short median survival of approximately 6 months even under conventional chemotherapy [24]. Currently, there is no standard chemotherapeutic regimen that is universally accepted for the frontline treatment of PEL. The lack of a consensus in treatment is mostly due to the low incidence of this disease and inability to conduct large-scale randomized studies to guide treatment and management decisions. However, some classical cytotoxic agents are considered as the mainstay of treatment. Other targeted therapeutic agents are also documented for clinical therapy in this patient setting and are described in Table 1.

The chemotherapeutic regimens for PEL mainly include CHOP (cyclophosphamide, doxorubicin, vincristine, and prednisone)-like chemotherapy and two intensive chemotherapy cocktails, dose-adjusted EPOCH (DA-EPOCH; etoposide, prednisone, vincristine, cyclophosphamide, and doxorubicin) and CDE (cyclophosphamide, doxorubicin, etoposide) [17,22]. The mechanisms of action of these cytotoxic agents include the inhibition of DNA, RNA, or protein synthesis except prednisone, which is a glucocorticoid receptor agonist that controls inflammation [48,49,50,51]. Specifically, doxorubicin and etoposide interact with topoisomerase II to inhibit DNA synthesis [52]; cyclophosphamide directly cross-links guanine bases in DNA double-helix strands to prevent DNA and RNA synthesis [53]; and vincristine stops tubulin dimer polymerization and subsequent microtubule formation by binding to the tubulin protein for inhibition of mitosis and protein metabolism [54]. Of interest, PEL usually displays more resistance to CHOP-based regimens than DA-EPOCH [22,55,56].

Several clinical studies assessing PEL clinical outcomes have found that the addition of other chemotherapeutic agents may improve the chemotherapy efficacy. Despite the lack of statistical significance, one study involving 28 PEL patients reported that the 5-year overall survival rate of patients receiving a CHOP-like regimen with high-dose methotrexate (MTX) was 45.7% compared to 34.4% of CHOP alone [57,58]. Another recent study involving 12 HIV-NHL patients (10 high-risk patients with DLBCL, including one EBV+, one EBV+/HHV-8+ PEL, and one unclassifiable NHL) reported that the response rate in HIV-NHL treated with vorinostat, a histone deacetylase inhibitor, combined with rituximab-based EPOCH was 100% (complete 83% and partial 17%), with a one-year event-free survival of 83% (95% confidence interval, 51.6%–97.9%), indicating that this novel combination regimen is tolerable and efficacious in patients with aggressive HIV-NHL [59], including those cases with oncogenic virus infection. Although there is progress in alternative combinations of chemotherapy, the advent of targeted therapies has also led to investigations in PEL.

Previous reports demonstrate favorable PEL patient outcomes following treatment with selective targeted therapeutic drugs. In a case report, bortezomib, an inhibitor of the 26S proteasome and nuclear factor B (NF-κB), in combination with doxorubicin and rituximab, substantially improved the performance of an elderly HIV-seronegative man of Mediterranean origin who was diagnosed with PEL [60]. Another study reported that intrapleural injection of bleomycin without any systemic therapy achieved a durable remission after pleurodesis in two HIV-seronegative patients with PEL [61]. Recently, a case report showed that after one month of lenalidomide treatment (a multi-functional agent with anti-angiogenic and anti-osteoclastogenic effects, and immunomodulatory activity), an HIV-seronegative patient with PEL significantly improved and after 18 months, complete remission persistence was progressively achieved [62]. Yet, another study reported that two PEL patients treated with oral rapamycin showed a positive clinical response and the authors postulated that KSHV+ patients who received a transplant could also benefit from substituting rapamycin for cyclosporine A and offer protection against PEL [63]. Thus, the addition of targeted therapies is sometimes necessary to increase chemotherapy efficacy in PEL patients, implicating potential pathways involved in mechanisms for chemotherapy resistance.

### 2.2. EBV-Associated Lymphomas

Lymphomas associated with EBV infection include three major B-cell malignancies: cHL, BL and DLBCL; NK/T-cell lymphomas; and two types of lymphomas in the immunocompromised patient setting, PTLD- and HIV/AIDS-associated lymphomas [64]. Similar to KHSV, EBV-associated lymphomas are traditionally treated with the same strategy as EBV-negative lymphoma counterparts, such as the anthracycline-containing chemotherapy (e.g., CHOP) as shown in Table 1. However, the status of EBV infections within lymphomas may also affect the option of treatment regimens.

cHL is a distinct clinical and pathological entity with heterogeneous genetic and virological features, with regards to EBV infection [65]. An epidemiological investigation showed that the pooled prevalence of EBV infection in cHL was about 47.9%, which is significantly higher in Africa and Central and South America than other regions [66]. Treatment for cHL usually involves combined chemotherapy, sometimes followed by radiotherapy. Typical treatments for advanced stage cHL include MOPP (mustargen, oncovin, procarbazine, and prednisone) [67], COPP (cyclophosphamide, vincristine, procarbazine, and prednisone), or ABVD (doxorubicin, bleomycin, vinblastine, dacarbazine) [68], whereas those that are PET (positron emission tomography)-positive are escalated to BEACOPP (bleomycin, etoposide, doxorubicin, cyclophosphamide, vincristine, procarbazine, prednisone), representing a more aggressive therapy that was initially developed in Europe [69]. In a recent report about an analysis of long-term survival in two randomized clinical trials, 1282 cHL patients received eight alternating cycles of COPP and ABVD (COPP/ABVD), eight cycles of bBEACOPP, or eight cycles of eBEACOPP in HD9; and 1670 patients received eight cycles of eBEACOPP or four cycles of eBEACOPP plus four cycles of bBEACOPP, plus consolidation radiotherapy to initial bulk and residual disease or no radiotherapy in HD12 (ClinicalTrials.gov NCT00265031), and strongly supported the use of eBEACOPP in advanced stage cHL [70]. However, one question remains in these clinical studies regarding the unknown percentage of cases that are EBV positive.

BL is an interesting mature B-cell NHL that has distinct features with endemic, sporadic, and immunodeficiency-associated variants [71]. The endemic BL is associated with EBV infection in over 95% of cases [72]. Historically, CHOP alone or in combination with methotrexate, cytarabine, ifosfamide, and/or etoposide is used for patients with BL in different clinical settings [72,73], but the addition of rituximab, an anti-CD20 chimeric monoclonal antibody, may improve the survival outcome for these patients [74,75]. Similar to the effectiveness of immunomodulatory agents in KHSV-PEL, recent in vitro evidence using BL and EBV lymphoblastic cell lines showed that pomalidomide increased the surface expression of immune markers and led to an enhanced susceptibility to NK cell-mediated cytotoxicity. This therapeutic approach has the potential to increase patients’ immune response to viral-associated tumors and improve patient outcome [76].

In contrast to BL, EBV infection is not as prevalent in DLBCL; however, when DLBCL tissues are EBV positive, these tumors are typically seen in the elderly and thought to be associated with chronic EBV infection as well as severe immunosuppression or immunosenescence. Treatments in the era of chemoimmunotherapy of EBV-associated DLBCL have similar guidelines with EBV-negative DLBCL, although EBV-positive DLBCL show more resistance and patients usually exhibit a lower response rate to chemotherapy than EBV-negative DLBCL [77,78]. Although, CHOP alone or in combination with rituximab (R-CHOP) is used for EBV-associated DLBCL patients. More recent data suggest higher rates of response to R-CHOP at overall response (OR) rates of 50% to 90% and complete response (CR) rates of 30% to 70% compared to CHOP at OR rates of 30% to 80% and CR rates of 30% to 50%, indicating the addition of rituximab has clearly improved survival outcomes for EBV+ DLBCL [79,80,81,82].

Extranodal NK/T-cell lymphoma, nasal type (ENKL) is sometimes associated with EBV infection, although the prevalence is dependent on extranodal involvement and geographic diversity in incidence. In clinical practice, patients with ENKL are usually resistant to CHOP-based regimens. The prominent reason for ENKL resistance to anthracycline-containing chemotherapies is most likely attributable to the expression of P-glycoprotein (P-gp), which is significantly linked to multidrug resistance (MDR) [83]. Thus, new chemotherapies avoiding the use of anthracycline are being developed and tested in prospective clinical trials and thus far have markedly changed the management of ENKL. In support of these positive clinical outcomes, l-asparaginase (l-asp) and/or non-MDR-related agents are now prescribed for newly diagnosed advanced ENKL patients. Alternatively, concurrent chemoradiotherapy as the first treatment tested in a qualified clinical trial setting for ENKL is also reported to improve clinical outcome when using radiotherapy in combination with classical chemotherapy, such as DeVIC (dexamethasone, etoposide, ifosfamide, and carboplatin) [84,85], VIPD (etoposide, ifosfamide, cisplatin, and dexamethasone) [86], VIDL (etoposide, ifosfamide, dexamethasone, and l-asp), MIDLE (methotrexate, ifosfamide, dexamethasone, l-asp, and etoposide), SMILE (steroid = dexamethasone, methotrexate, ifosfamide, l-asp, and etoposide) [87,88], and GELOX (gemcitabine, l-asp, oxaliplatin) [89]. Recently, a study combined both of the new therapeutic strategies to treat adult ENKL with polyethylene glycol-conjugated asparaginase, and CHOP in combination with radiotherapy showed high overall survival (OS) rates of 100%, 90.61%, and 80.54% at 1, 2, and 3 years, respectively [90]. Although EBV-DNA sero-levels were not measured during this study, these regimens may also significantly benefit patients with EBV-associated ENKL. Moreover, a clinical study showed that the LOP regimen (l-asparaginase, vincristine, and dexamethasone) in combination with intensity-modulated conformal radiotherapy for nasal ENKL patients resulted in a higher overall remission rate, survival rate, and lower adverse reactions [91].

As mentioned above, EBV infection is also associated with two types of lymphomas in the immunocompromised patient setting. In AIDS-associated lymphomas, conventional chemotherapy in combination with combined antiretroviral (cART) therapy is required since antiretroviral therapy alone is inadequate for the treatment of the lymphoma [92]. For PTLDs, CHOP-like chemotherapy still remains the only option for many patients with aggressive PTLDs [26].

## 3. Involvement of KSHV-Encoded Proteins into Drug Resistance of Lymphomas

**Figure 1:**
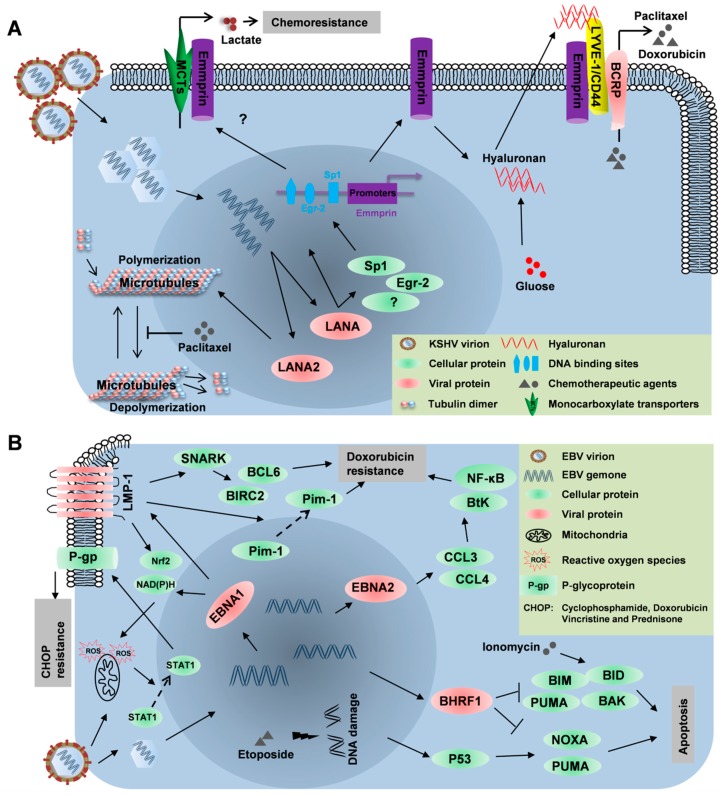
The schematic of oncogenic virus-mediated chemoresistance in lymphomas. (**A**) The downstream chemoresistance pathways induced following B-cell infection with the Kaposi’s sarcoma associated herpesvirus (KSHV) driven by the LANA and LANA2 viral proteins. (**B**) The downstream chemoresistance pathways induced following B-cell infection with the Epstein–Barr virus (EBV) facilitated by the EBNA1, EBNA2, and BHRF1 EBV-encoded proteins.

Various host and viral factors influence the sensitivity of virus-associated lymphomas to anticancer drugs. Despite overloading patients with cytotoxic chemotherapeutic agents to trigger cellular stress that should culminate in death of the malignant cells, it is impossible to eliminate oncogenic viruses in these patients. For instance, PEL cell lines display high heterogeneity in the drug response [93], and some recent studies found that this chemoresistance was promoted by the presence of specific viral proteins, particularly LANA and LANA2 (summarized in Figure 1A).

### 3.1. LANA

LANA is a master regulator of KSHV latency, and primarily functions to maintain latency, enable episome DNA replication and segregation, and regulates gene transcription. LANA has also been identified as an oncoprotein to steer immune evasion and facilitate tumorigenesis [94,95]. Our recent work highlighted an extended role of LANA in PEL chemoresistance [40,96]. We discovered that LANA contributed to PEL resistance to paclitaxel and doxorubicin by regulating the expression of Emmprin (Basigin; CD147), a multifunctional glycoprotein belonging to the immunoglobulin superfamily. This protein is involved in interactions with two key MDR cell surface proteins: A hyaluronan receptor known as the lymphatic vessel endothelial hyaluronan receptor-1 (LYVE-1) and a drug transporter ABCG2/BCRP [39,96]. In support, targeting Emmprin, LYVE-1, or BCRP by RNA interference promoted PEL cell apoptosis induced by chemotherapy while the overexpression of Emmprin greatly enhanced the resistance of chemosensitive tumor cells to paclitaxel and doxorubicin [39,96]. The mechanism for LANA-induced Emmprin is not fully understood, although conceivably, LANA may bind directly to the promoter of Emmprin or indirectly as a complex with other cellular transcription factors, such as Sp1 and Egr-2, for which the promoter of Emmprin contains putative binding sites [96,97]. Previous studies demonstrated that the drug resistance of malignant cells could be related to glycolysis and lactate secretion that require monocarboxylate transporters (MCTs) to efflux lactate resulting from glycolysis [98,99]. Interestingly, Emmprin interacted with various MCTs, and PEL cells preferentially utilize glycolysis as an energy resource for survival [100,101]. Therefore, further studies are of interest to determine whether Emmprin regulation of MCTs functions and lactate efflux may impact PEL chemoresistance.

### 3.2. LANA2

LANA2, a nucleocytoplasmic shuttling protein, is exclusively expressed within KSHV-infected B cells as a B-cell-specific latent protein [102]. Although LANA2 may not be detected using serums of KSHV+ patients [102], it still plays an important role in KSHV-induced tumorigenesis. Previous studies identified its oncogenic abilities based on the supportive data that LANA2 inhibited p53- and PKR-dependent apoptosis [102,103], and also inhibited virus-mediated transcriptional activity of interferon alpha promoter [104]. Meanwhile, LANA2 interacted with the pocket proteins, pRb, p107, and p130, and inhibits their sumoylation to regulate cell-cycle arrest [105]. Moreover, LANA2 inhibited PML-mediated transcriptional repression of the survivin gene by disrupting PML oncogenic domains [106]. Interestingly, a previous study found that LANA2 induced paclitaxel resistance of PEL cells through binding to polymerized microtubules to prevent microtubule stabilization caused by paclitaxel [41].

### 3.3. Other Viral Proteins

One study found that KSHV infection induced AKT hyperphosphorylation, bortezomib-resistance, and GLUT-1 plasma membrane exposure in a THP-1 monocytic cell line [107]. Moreover, KSHV latent infection in bladder cancer cells promoted drug resistance by reducing reactive oxygen species (ROS) [108]. However, it remains unclear which viral proteins are responsible for these observations. Unexpectedly, KSHV-encoded vFLIP expression conferred selective chemosensitization to bleomycin-mediated cytotoxicity in HEK293 cells [109]; however, the influence of vFLIP in PEL chemoresistance remains unclear.

## 4. Involvement of EBV Components into Drug Resistance of Lymphomas

EBV is not only an important pathogen etiologically related to many human cancers but also a considerable barrier to chemotherapy for EBV-associated malignancies. Previous reports indicated latent EBV infection interfered with the responses of BL-derived cells to cytotoxic drugs by activating the mitotic spindle assembly checkpoint [110]. Moreover, EBV infection may confer chemoresistance of gastric carcinoma in response to the chemotherapeutic agents, 5-Fluorouracil and docetaxel [111,112]. Although the underlying mechanisms are still unclear, some data suggest the contribution of EBERs to chemoresistance in EBV-associated gastric cancer cells [113]. Here, the roles of EBV components in the chemoresistance of virus-associated lymphomas are summarized (Figure 1B).

### 4.1. LMP1

LMP1, a member of the tumor necrosis factor receptor superfamily and constitutively active membrane protein, is considered as the principal viral oncoprotein critical for immortalization and transformation of B cells through mimicking the CD40 receptor signaling pathway [114,115,116]. Some studies suggest the involvement of LMP1 in chemoresistance to treatment for EBV-associated lymphomas. For instance, a study revealed that LMP1 conferred drug resistance of T-cell lymphoma cell lines in response to doxorubicin by upregulating the expression of SNF1/AMP kinase-related kinase, which subsequently increased the expression of anti-apoptotic factors, BCL6 and BIRC2 [43]. Another group reported LMP1-mediated chemoresistance for doxorubicin was also associated with an oncogenic gene, *Pim-1*, since LMP1 enhanced the expression of Pim-1 and translocated Pim-1 to the cytoplasm. This mechanism could lead to an increase in tumor cell survival [44]. In addition, LMP1 was found to mediate EBV-transformed B cell resistance to apoptosis and DNA damage through induction of autophagy [45,46,117,118]. For instance, there was a constitutively high level of autophagy in these apoptosis-resistant cell lines (mostly DLBCL and PTLD), which express high levels of the pro-autophagic protein, BECN1/Beclin 1, based on the activation of NF-κB signaling by LMP1 [45]. Another recent study reported that LIMD1 (LIM domain-containing protein 1) was induced and protected EBV-transformed cells from DNA damage-induced cell death through LMP1-activated IRF4 and NF-κB signaling and autophagy [46]. However, the clinical relevance of chemoresistance mediated by EBV/LPMP1-activated autophagy, as well as the translational potentials (e.g., autophagy inhibitors), still requires further investigations.

EBV-associated NK/T-cell lymphomas are highly resistant to CHOP, probably due to upregulation of P-gp, an energy-dependent efflux pump that excretes drugs from the cytoplasm to the extracellular space [119,120]. High expression of P-gp is also connected with ROS production, which is induced by EBV infection [121,122]. Previous work established that induction of intracellular ROS production under EBV latent infection elevated P-gp expression via the STAT1 pathway while downregulation of ROS by NecroX-5, an ROS scavenger, effectively attenuated P-gp-associated chemoresistance in EBV-positive NK/T-cell lymphoma [123]. These findings indicate ROS may represent a potential therapeutic target for reducing chemoresistance in this subtype of EBV+ lymphoma. Moreover, recent studies demonstrated that LMP1 and/or other viral components were also involved in P-gp dependent chemoresistance. LMP1 promoted excessive ROS production from EBV-related malignant cells through activation of NAD(P)H oxidases and induction of nuclear factor erythroid 2-related factor 2 (NRF2) [124,125]. Other studies showed that the expression of another EBV-associated protein, EBNA1, in either latent or lytic phases, regulated LMP1 expression [126], and upregulated cellular antioxidant defense to enable B-cell growth, transformation, and immortalization [122].

### 4.2. EBNA2

EBNA2 is one of the six viral nuclear proteins expressed in latently infected B lymphocytes [30]. EBNA2 is thought as the master regulator for viral latent transcripts and numerous host genes, and is required for the transformation and immortalization of EBV-infected cells [5]. EBNA2 expression in EBV-infected B lymphocytes is critical for lymphoblastoid cell growth and survival. An interesting report showed the involvement of EBNA2 in the doxorubicin resistance of EBV-associated B lymphoma cells occurred through increasing CCL3 and CCL4, which in turn activated the BTK and NF-κB pathways [42].

### 4.3. BHRF1

Another EBV-encoded protein, BHRF1 (BamHI-H rightward open reading frame 1), is a functional homologue of the human BCL2 anti-apoptotic protein. Overall, the levels and interactions between pro-apoptotic and pro-survival protein family members decide the fate of the intrinsic/mitochondrial apoptotic pathway and whether a cell will undergo apoptotic cell death [5]. Expression of BHRF1 in EBV infection is an important regulator that blocks host cell apoptosis and death by sequestering cellular pro-apoptotic proteins, such as BIM [127]. This function was correlated in a recent report to BHRF1 promoting chemoresistance in BL cells [45]. BHRF1 was shown to inhibit multiple cellular pro-apoptotic proteins, including BIM, BID, PUMA, and BAK, and prevent BL tumor cell apoptosis to etoposide and ionomycin. Loss-of-function assays using BHRF1 mutants in EBV-infected B-cell lymphomas also supported its role in chemoresistance and indicated that BHRF1-mediated anti-apoptosis was associated with mutants of G99A, R100D, and F72W [45]. The authors also found that one of the mutants, F72W, exhibited different effects on the response to etoposide and ionomycin. This differential effect may be due to the alternative mechanisms for inducing apoptosis specific to each chemotherapy agent. Ionomycin acts primarily through Bim [128] while etoposide activity is dependent on the activation of TP53 and concomitant upregulation of PUMA and NoxA [129].

## 5. HTLV-1

Human T-cell leukemia/lymphoma virus type 1 (HTLV-1) is a retrovirus that causes adult T-cell leukemia/lymphoma (ATLL), an aggressive form of T-cell malignancy with four major subtypes: A smoldering type, chronic type, lymphoma type, and leukemic type [130]. One of the viral proteins, Tax, is designated as the master modulator in contribution to HTLV-1-associated leukemogenic. Currently, the common first-line chemotherapies used to treat ATLL are the same as those used to treat other types of T-cell lymphomas, including CHOP, CHOEP (cyclophosphamide, doxorubicin, vincristine, etoposide, and prednisone), DA-EPOCH, and Hyper-CVAD (cyclophosphamide, vincristine, doxorubicin, and dexamethasone) [131]. Although ATLL displays intrinsic chemoresistance, the contribution of this oncogenic virus or viral proteins to tumor cell chemoresistance remains unknown.

## 6. Conclusions

In virus-associated lymphomas, the presence of viral proteins increases the complexity of the tumor microenvironment and induces the expression of oncogenes that can limit therapeutic efforts. Accumulating data demonstrate the involvement of viral components in multidrug chemoresistance of lymphoma cells and several mechanisms are potentially associated with oncogenic virus-mediated chemoresistance: (1) A reduction of drug accumulation in lymphoma cells by regulating the activities of cellular drug transporters, (2) the hijacking of chemotherapeutic agent cellular targets, (3) the regulation and/or interaction with key factors involved in chemo-induced cell death responses, and (4) the antagonizing of the anti-cancer effects of chemo-drugs by inducing oncogenic signaling during infection. Therefore, infection with oncogenic viruses clearly contributes to lymphomagenesis and the pathology of these diseases in addition to compromising the efficacy of chemotherapy. However, since to date the majority of these findings are derived from in vitro studies, the question remains of whether these identified and postulated mechanisms for viral-driven chemoresistance are the same as those that occur within the tumor of a patient. There are many challenges for successful treatment of virus-associated lymphomas that center around the inability to tease out the oncogenic activity of the virus and eradicate the virus itself. Most oncogenic viruses involved in the development of lymphomas are lacking vaccines or antiviral drugs to prevent their infections. Similarly, current antiviral drugs do not effectively target viruses during latent infection, especially for those oncogenic herpesviruses. As well, targeting the tumor proves difficult in the setting of virus infection since the mechanisms behind oncogenic virus-mediated chemoresistance still remain largely unclear. Furthermore, many patients with virus-associated lymphomas are immunosuppressed and/or immunodeficient, and these patients are already dose sensitive. Chemotherapy, especially high-dose treatments that are often necessary for this patient population, may cause additional severe toxicity and other side effects. Lastly, some chemo-drugs may induce viral lytic reactivation and facilitate virus dissemination. Overcoming these obstacles may help to improve the chemotherapy efficacy and prolong the survival time of patients with virus-associated lymphomas.

## Figures and Tables

**Table 1:** Oncogenic virus-encoded proteins are involved in the chemoresistance of lymphoma cells based on in vitro studies.

Viruses	Transcripts	Drugs/Agents	Lymphomas	Mechanisms	Ref.
KSHV	LANA	Doxorubicin	PEL	Emmprin, LYVE-1, BCRP and hyaluronan signaling	[39,40]
		Paclitaxel	PEL	Emmprin, LYVE-1, BCRP and hyaluronan signaling	[39]
	LANA2	Paclitaxel	PEL	Modulating microtubule dynamics to prevent microtubule stabilization induced by paclitaxel	[41]
EBV	EBNA2	Doxorubicin	DLBCL	Regulating CCL3 and CCL4-mediated activation of NF-κB and Btk signaling	[42]
	LMP1	Doxorubicin	T-cell lymphoma	Upregulating SNARK to increase expression of anti-apoptotic genes, such as BCL6 and BIRC2; Enhancing expression of Pim-1 and translocating Pim-1 to the cytoplasm	[43,44]
		Nutlin-3	DLBCL/PTLD	Upregulating pro-autophagic proteins such as Beclin 1 and Sestrin 1 through LMP1-activated NF-κB signaling	[45]
		Ionomycin	BL	Upregulating LIMD1 through LMP1-activated IRF4 and NF-κB signaling as well as autophagy	[46]
	BHFR1	Etoposide	BL	Interacting with the cellular pro-apoptotic BCL-2 proteins, BIM, BID, PUMA and BAK, to regulate apoptosis	[47]
		Ionomycin			
